# Gemmological, Spectroscopic, and Origin Description Studies of Tourmaline from Yunnan, China

**DOI:** 10.3390/molecules30183680

**Published:** 2025-09-10

**Authors:** Qishen Zhou, Fangmin Zhan, Haochi Yu, Zhuo Lu, Xin Wan

**Affiliations:** 1 Gemological Institute, China University of Geosciences, Wuhan 430074, China; zqs@cug.edu.cn (Q.Z.); zfmwcy@cug.edu.cn (F.Z.); wanxin@cug.edu.cn (X.W.); 2 School of Earth Sciences, China University of Geosciences, Wuhan 430074, China; 3 School of Jewelry, West Yunnan University of Applied Sciences, Tengchong 679100, China

**Keywords:** tourmaline, chemical component, spectroscopic analysis, gemological characteristics, Yunnan, China

## Abstract

The Nujiang region of Yunnan is by far the richest tourmaline-producing mining area in China. Since the discovery of the tourmaline-bearing deposit in Yunnan Province in 1980, there have been few comprehensive gemmological studies of this deposit. Therefore, the results of tests on 32 tourmaline samples from the Fugong and Gongshan regions of Yunnan are reported in this paper. The chemical composition of the Yunnan tourmalines was analyzed, and the contents of major trace elements were compared with those of tourmaline samples from different localities reported in the literature to highlight their specific provenance characteristics. Microscopic observation revealed the presence of liquid, gas, and solid inclusions; Raman spectra indicated the presence of constitutional water and CH_4_-C_2_H_6_ dihydrate in the Yunnan tourmalines and also pointed to the influence pattern of the Fe content. The infrared spectrum in the range of 4000–4800 cm^−1^ showed the frequency of metal cations and hydroxyl groups. Based on the characteristic peaks at 4343 cm^−1^ and 4600 cm^−1^, a quick distinction between elbaite and dravite could be made. UV–Vis absorption spectroscopy analysis showed that in yellow tourmalines, Mn^2+^-Ti^4+^ IVCT is the main cause of color, while green coloration occurs due to Fe^2+^–Fe^3+^ interactions or Cr^3+^ and V^3+^, and the pink color is caused by Mn^3+^ d-d transitions. The three-dimensional fluorescence spectra revealed the presence of the main fluorescence peaks at λex280/λem320 nm and λex265/λem510 nm in the tourmaline samples analyzed and the fluorescence intensity with Ti and Fe contents.

## 1. Introduction

Tourmaline is one of the most appreciated gemstones on the market, valued among consumers for its high-quality luster, rich color, and unique polychromatic nature. Tourmaline is a complex borosilicate with significant compositional variability, resulting in a wide range of distinct mineral species [1]. In accordance with Henry et al. [2], the general formula of tourmaline can be written as XY_3_Z_6_(T_6_O_18_)(BO_3_)_3_V_3_W. Tourmaline is a multicolored gemstone, exhibiting different colors depending on chemical composition and crystal structure [3,4,5].

**Figure 1:**
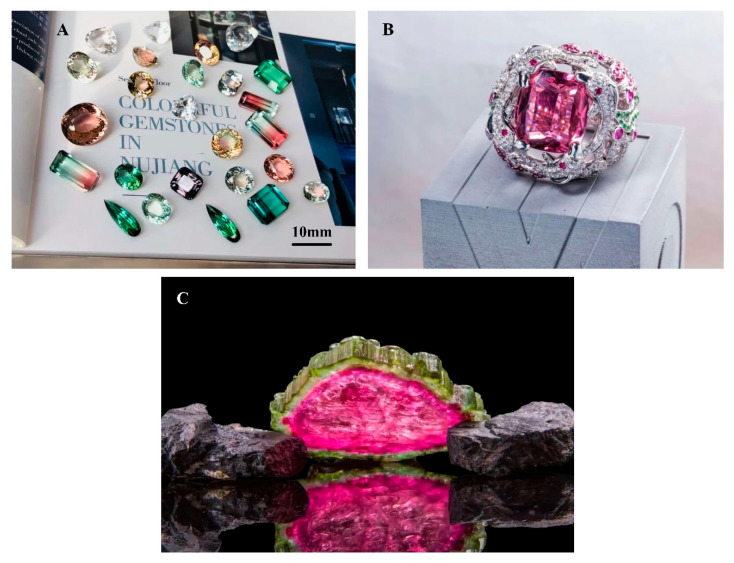
(**A**) High-clarity faceted tourmalines of rich color from the Nujiang region, Yunnan Province; scale bar = 10 mm. (**B**) The size of the ring is 26.6 × 24.8 × 31.8 mm. The photo was obtained from the Lan York Tourmaline Museum, which shows a single 26.88 ct natural pure red tourmaline, known as “Sulamima”, meaning the goddess of flowers, from a legendary story of the Nujiang River. (**C**) Natural watermelon tourmaline from the Nujiang region of Yunnan Province, known locally as “tourmaline candy”. The tourmaline crystal is 23 cm long, 17 cm at its widest point, and 1.5 cm thick on average; it weighs 877 g. Photo: Lan York Tourmaline Museum.

Tourmaline from the Nujiang region of Yunnan, China, is little known in the gemmological literature. At present, there are two major tourmaline-producing areas in China: one in the Altai region of Xinjiang, which is underexploited to date, and one in China’s southwestern border area, in the Nujiang region of Yunnan Province, which is still actively mined. At the time of its discovery, the richly colored, high-quality tourmalines that dotted the banks of the Nujiang River were remarkable (Figure 1).

Tourmaline is a borosilicate that crystallizes in the trigonal crystal system. Its structure consists of six-membered silicate rings with the complex chemical formula XY_3_Z_6_(T_6_O_18_)-(BO_3_)_3_V_3_W, in which X=Na^+^, Ca^2+^, K^+^, or □ (Vacancy); Y=Fe^2+^, Mg^2+^, Mn^2+^, (Al^3+^, Li^+^), Fe^3+^, Cr^3+^, or V^3+^; Z=Al^3+^, Fe^3+^, Mg^2+^, Cr^3+^, V^3+^; T=Si^4+^, Al^3+^, B, or Be; V=O^2−^ or OH^–^; and W=O^2−^, OH^–^, or F^–^. In previous reports [6,7,8,9], the gemmological properties of single-origin, single-species tourmaline (lithium tourmaline and magnesium tourmaline) were studied. Tourmaline produced in Yunnan, China, contains both elbaite and magnesium tourmaline, which are of great value for the identification of producing areas and for the study of different varieties. Zhong (2014) speculated that the black inclusions within tourmaline are solid inclusions filled with organic matter and iron [10]. Hoang et al. (2011) utilized the differences in elemental concentration in paraíba tourmalines to confirm their origin [11]. Abduriyim et al. (2016) and Okrusch et al. (2016) also investigated trace element differences among different geographical origins of Paraiba tourmaline [5,6]. Laurs et al. (2007) established a theoretical and experimental basis for the distribution of Mn and Ti contents, which are the main elements responsible for the color of yellow tourmaline [12]. Prasad (2005) observed three fundamental vibrations at 3730–3720 cm^−1^, 3637–3627 cm^−1^, and 3550–3500 cm^−1^ in tourmaline samples [13]. Suwanmanee et al. (2021) studied variations in the absorption spectrum at 520 nm produced by Mn^3+^ in pink tourmaline irradiated at different intensities [14]. Previous research has analyzed O-H characteristic peaks and inclusions of tourmaline using infrared spectroscopy [13]. Hoang et al. (2011) and Watenphul et al. (2016) employed Raman spectroscopy to study the [SiO_4_] tetrahedra of tourmaline and to analyze the occupancy of elements at the Y and Z sites [11,15].

In this study, 32 tourmaline samples representing different colors from Yunnan Province were selected for a systematic study including conventional gemmological characterization, chemical composition, and spectroscopic analyses. Specifically, the gemmological properties were evaluated; the chemical composition was measured by LA-ICP-MS and compared with data from tourmalines of different localities in order to highlight their provenance characteristics; UV-Vis, infrared, and Raman spectroscopy were employed to identify color-causing agents for the observed colors. The aim of this study is to provide compositional and spectroscopic characteristics of gem-quality tourmalines from Yunnan Province (China). The result obtained can be applied for origin identification.

### 1.1. Location and Access

**Figure 2:**
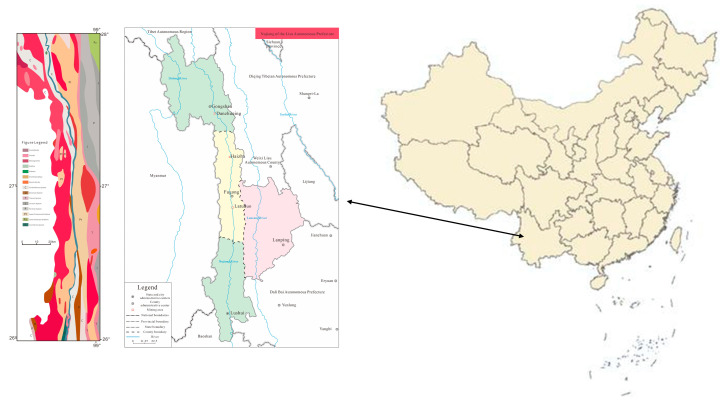
The three counties, Gongshan, Fugong, and Lushui, along the banks of the Nujiang River, host the main tourmaline mines of Yunnan Province. The regional geology and rock types that characterize the Nujiang Prefecture are presented in the right panel. Tourmaline is mainly produced from granite near river banks. The image was drawn by Yu.

The Nujiang Lisu Autonomous Prefecture is located in the northwestern part of Yunnan Province. Lushui, the capital city of the Nujiang Lisu Autonomous Prefecture, is located to the south of Fugong and Gongshan counties, along the Grand Canyon of the Nujiang River, in the western region of Yunnan Province. Specifically, Fugong and Gongshan counties are important tourmaline production areas (Figure 2). Fugong County is located in the middle of the Hengduan Mountains in northern Yunnan, at the intersection of the Biluo Snow Mountain and the Gaoligong Mountain, in the center of the Grand Canyon of the Nujiang River. This canyon runs from north to south across the Nujiang Prefecture, forming a narrow “V”-shaped valley. Fugong County’s highest point is the peak of the Biluo Snow Mountain Gala beat, 4379 m above sea level, whereas the lowest point for the junction with Lushui City is the surface of the Nu River, 1010 m above sea level. Given the relative height difference of 3369 m, it is a typical alpine canyon mountain landscape. Gongshan County is located between three mountains and two rivers, with the highest peak at 5128 m and the lowest point at 1170 m, and with a height difference of 3958 m. Three main mining areas can be reached by car from downtown Lushui, namely Latuduo mine and Haizha mine, located to the south and north of the Fugong County, respectively, and Danzhuquing mine in Gongshan County, which is the farthest from Lushui City.

### 1.2. Geology

**Figure 3:**
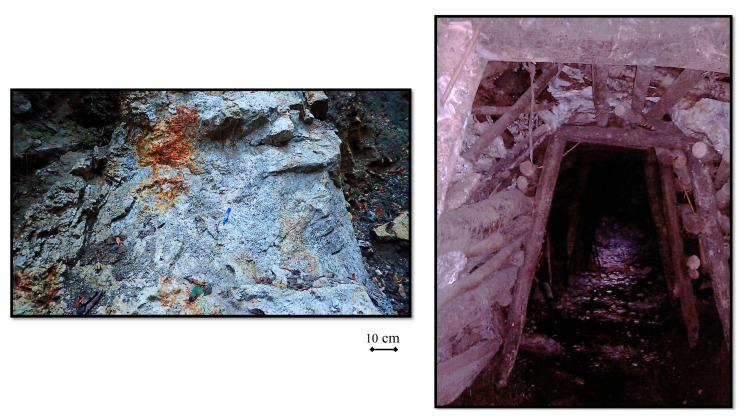
The left panel shows some pegmatitic veins hosting black tourmalines and feldspar crystals. The right panel shows a tourmaline mining adit in Yunnan Province.

The mining areas are located in the Fugong and Gongshan counties, scattered on both sides of the Bangong-Nujiang Suture Zone. The basement rocks consist of the metamorphic rock systems of the Gaoligong Mountain Group and the Chongshan Rock Group (early meta-paleogene), which are exposed straddling the Nujiang River between the Gaoligong Mountain and the Biluo Snow Mountain. The rocks of the basement were strongly deformed and metamorphosed, showing complex tectonic deformations, and the intensity of metamorphism is variable [16]. It has the characteristics of deep deformation and metamorphism, and generally, there is more intense metamorphism. Inside of the deep metamorphic rock zone, a large-scale ductile shear zone mainly composed of mylonite develops. The Late Paleozoic sedimentary rocks, which are distributed along the Nujiang River Gorge and the Dulong River Gorge, were subjected to strong extrusion and deformation and regional phenomena metamorphism. In particular, the Late Paleozoic rocks, which are exposed in the Nujiang Gorge, form a complex back-slope structure (Figure 3). High-quality tourmalines are mainly hosted in the metamorphic rock systems of the Gaoligong and Chongshan Groups. Granite and basal ultramafic rocks occur intermittently in several formations during different geologic periods, especially in the silica-enriched rocks of the Himalayan tectonic period.

### 1.3. Mines and Production

The Nu River merges with the Lancang and Jinsha Rivers, forming the famous “Three Rivers” area. The region comprises southern Qinghai, eastern Tibet, western Sichuan, and western Yunnan, as well as the eastern Qinghai–Tibet Plateau and the western Yunnan–Guizhou Plateau, covering an area of about 500,000 square kilometers. Fault structures in the Sanjiang area are well developed, most of which are dominated by the Jinshajiang–Ailaoshan fault zone, Lancang River fault zone, and Nujiang fault zone. In this region, magma movement occurs frequently, and intrusive rocks and volcanic rocks are exposed on a large scale, forming multi-stage magmatic rocks, of which meso-primary magmatic rocks are the majority type, forming a compound batholeptic, basic-ultrabasic rock belt and a volcanic rock belt with a large area. The above magmatic rock belt extends from the south to north and is parallel to the Jinshajiang and Bangong-Nujiang suture zones in a spatial structure. It constitutes a unique geological structure landscape in the Sanjiang area. The three most important tourmaline-bearing deposits in Yunnan Province are the Latuduo mine and the Haizha mine, located in Fugong County, and the Danzhuquing mine in Gongshan County. The Fugong Latuduo tourmaline mining area is located in the Latuduo village, after which it is named. The rock system exposed in this area is mostly metamorphic and belongs to the Chongshan Group. The tourmaline crystals are mainly hosted in miarolitic cavities within quartz–feldspar pegmatite bodies. The colors of such tourmalines are green, pink, and yellow. The Fugong Haizha tourmaline mining area is located in the Shiyue Township, on the west bank of the Nujiang River. The rocks exposed in this area are represented by gray and dark gray thin- to medium-thick layered slate, gneiss, and marble (Figure 3).

Gem-quality tourmalines occur in miarolitic cavities within granitic rocks. The tourmaline crystals of the Haizha mine are characterized by different colors, including dark green, green, light green, and yellow-green. The Danzhuquing mine in the Gongshan County is an alluvial deposit consisting of residual quartzite and pegmatitic rock fragments. The tourmaline crystals appear as detrital grains, which are mostly green in color with a small amount of light blue coloration.

The Danzhuquing mine was the first place in Yunnan where tourmaline was found in the 1950s and 1960s [17]. The tourmaline-bearing sediments were eroded and moved along with rock avalanches or mudslides. In the 21st century, as the commercial value of tourmaline increased, mining activities became more intense.

## 2. Results and Discussion

### 2.1. Basic Gemological Features

All samples analyzed in this study are from Yunnan Province (China), including round-cut and stepped-cut facets as well as unpolished rough stones. The colors include yellow, pink, dark green, and light green, with a vitreous luster. The density range of the samples was calculated by using the hydrostatic weighing method: 2.85–3.18. The refractive indices were measured using a gem refractometer and ranged from 1.615 to 1.645, with a birefringence of 0.019–0.022 and a monoclinic crystal negative luminosity. The approximate refractive index of the unpolished tourmaline sample was measured by spot reading to be 1.64. By rotating the gemstone under a crossed polarizer for one round, the phenomenon of four times light and dark alternations is shown, which indicates that the gemstone has a non-homogeneous character. Under the dichroic microscope, the green tourmaline shows light green and yellow-green coloration, indicating that the gemstone has strong dichroism. The sample is mostly non-fluorescent at UV long wavelengths (365 nm) and partially fluoresces in dark yellow at short wavelengths (254 nm). The needle-like inclusions, gas–liquid two-phase inclusions, and some visible solid particle inclusions are visible under the microscope or under tenfold magnification using transmitted light irradiation.

### 2.2. Microscopic Features

Tourmaline gemstones from Yunnan Province are rich in inclusions, which vary by mine; they are mostly solid inclusions, while the rest are two-phase (liquid–gas) fluid inclusions. Tourmaline samples from Fugong and Gongshan were selected to observe their microscopic characteristics.

**Figure 4:**
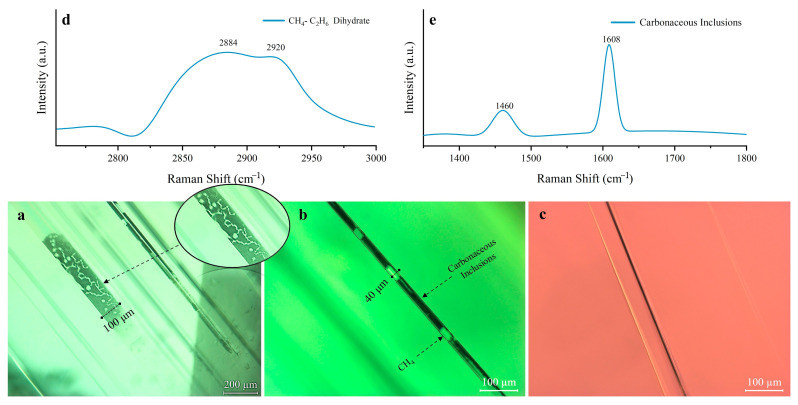
Typical inclusions in the tourmalines from Yunnan. (**a**,**b**) show regularly arranged tubular and black inclusions and gas–liquid inclusions within the tubes. (**c**) shows a tubular inclusion without carbonaceous material. (**d**,**e**) show the Raman spectra of the inclusions. The black inclusions are presumed to be organic matter (**e**), whereas the gas–liquid inclusions are presumed to be CH_4_-C_2_H_6_ (**d**). Photographs were taken by Yu, Li.

Tubular inclusions are common in the Yunnan tourmalines (Figure 4), occurring both in clusters and as individual growth tubes, with a wide variation in length and diameter. The length runs the entire length of the stone and is about 3–5 mm, with an average diameter of 30–40 μm [18]. The tubes are filled with black inclusions containing bubbles inside (e.g., Figure 4a,b). The two-phase (liquid–gas) inclusions are tested by Raman spectroscopy (Figure 4d). According to Zeng (2016), the Raman peaks at 2919 and 2888 cm^−1^ are attributable to the presence of CH_4_ and ethane, respectively, which produce a Raman shift depending on the presence of aqueous compounds [19]. Therefore, it is speculated that the two-phase (liquid–gas) inclusions appearing in the tourmaline samples studied is CH_4_-C_2_H_6_. The black inclusions filled in the tubes are presumed to be asphalt, and Raman vibrations in the range of 1300 cm^−1^–1600 cm^−1^ are often present in carbonaceous organic matter (Figure 4e), which is related to the stretching vibration of carbon–carbon double bonds [20,21].

Some of the tubular inclusions are colorless and transparent (Figure 4b). Mineral grains are wrapped in the terminal part of tubular inclusions, probably because small crystals of tourmaline crystallized first in the early solution and individual sulfides were captured during the growth of tourmaline host crystals. This condition induced the formation of elongated tubular inclusions, which have both theoretical and practical significance for studying the formation of inclusions [22]. When tubular inclusions are oriented, they are responsible for chatoyancy and cat’s eye effects after polishing by cutting and grinding the gemstones.

**Figure 5:**
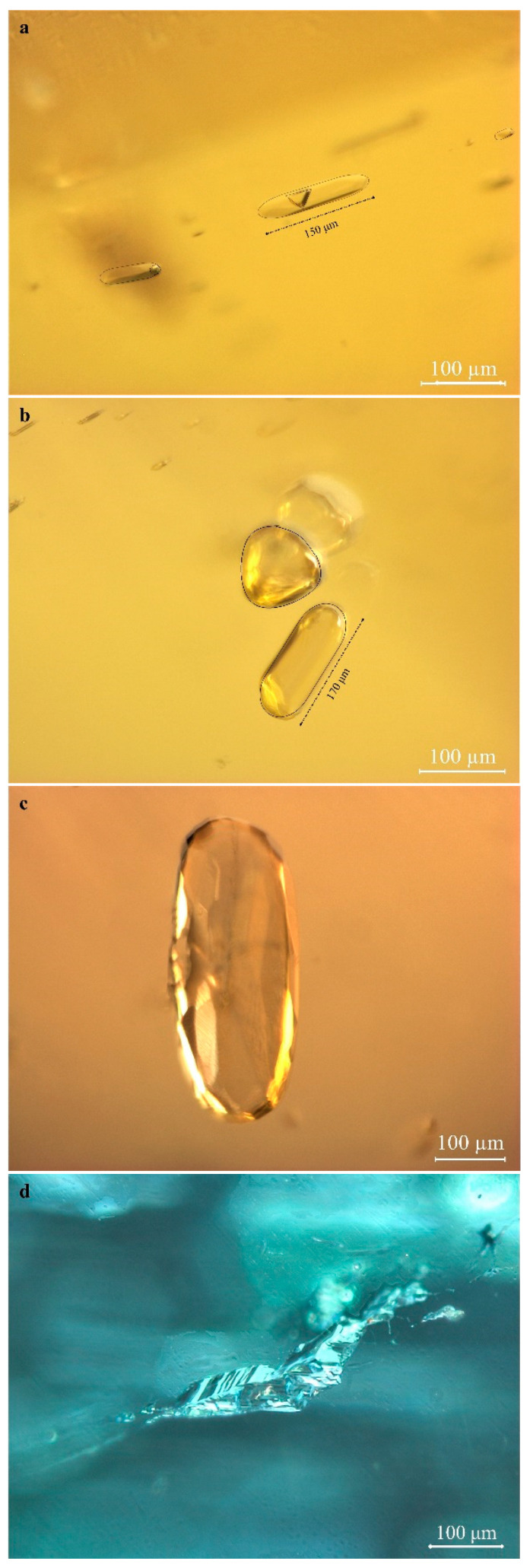
Gas–liquid inclusions and solid inclusions within the tourmalines from Yunnan Province. (**a**–**c**) show an elliptical gas–liquid inclusion, and (**d**) is a negative inclusion. Photographer: Yu.

Gas–liquid and liquid–solid two-phase inclusions or gas–liquid–solid three-phase inclusions are present within the tourmaline samples studied, with a small number per unit area and a large volume of 100 μm or more. Such inclusions can be subelliptical, long columnar, or geometric in outline and mostly irregular. Oval droplet-shaped inclusions (Figure 5a–c), triangular solid or rounded gas inclusions (Figure 5a), and negative crystals (Figure 5d) are common and appear distinctly reflective when illuminated. These inclusions are often accompanied by healed fissures extending to the tourmaline surface.

### 2.3. Chemical Element Analysis

**Figure 6:**
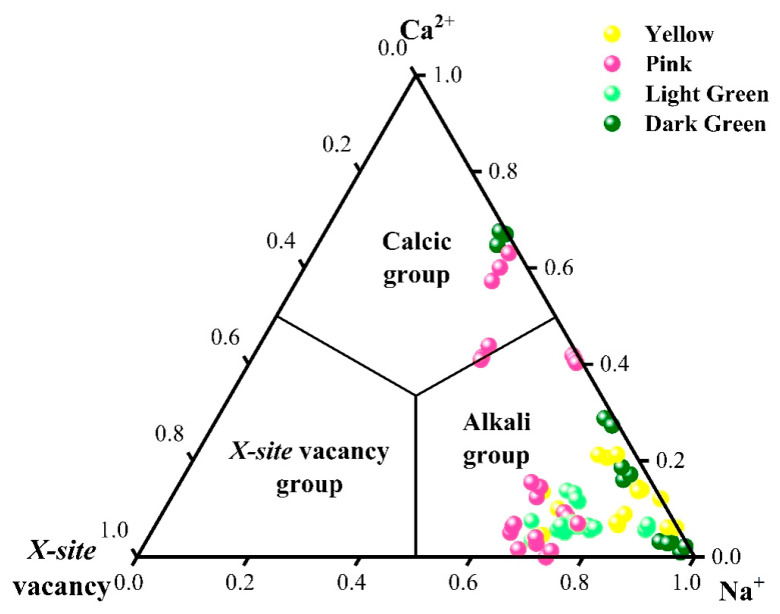
X-site ternary plot. Each point represents the composition of a single sample determined by LA-ICP-MS analysis. The different colored samples are distinguished by colored labels. Data show that most of the tourmalines analyzed belong to the alkali group.

According to the dominance of (Na + K), Ca, and vacancies at the X-site, the tourmaline samples studied can be classified as alkali, calcic, and X-site vacant [23]. These three types of tourmaline can be classified in a ternary plot (Figure 6). From the X-site ternary plot, it can be pointed out that most of the tourmalines analyzed contain high Na^+^ contents lying in the alkali group field. However, some of them, especially some of the pink and the dark green ones, occupy the calcic compositional field. All the light green and yellow tourmalines belong to the alkali group.

Thirty-two tourmalines from the Fugong and Gongshan counties in Yunnan Province are of both elbaite and dravite composition (Figure 6), with elbaite predominating, and the calculated mean empirical formula for the yellow elbaite is ^X^(Na_0.7690_Ca_0.0617_K_0.0035_□_0.1657_)^Y^(Li_1.0678_Fe_0.0018_Mn_0.4683_Al_1.8297_)^Z^(Al_6_)^T^[Si_5.9611_Al_0.0389_O_18_]^B^[B_0.8652_□_0.1348_O_3_]_3_^V^(OH)_4_. The mean empirical formulae of pink elbaite samples is ^X^(Na_0.6832_Ca_0.0633_K_0.0028_□_0.2507_)^Y^(Li_1.2107_Fe_0.0912_Mn_0.2503_Al_1.6344_)^Z^(Al_5.5075_Si_0.4925_)^T^[Si_5.1596_B_0.8404_O_18_]^B^[BO_3_]_3_^V^(OH)_4_. Green elbaite: ^X^(Na_0.8710_Ca_0.0511_K_0.0024_□_0.2904_)^Y^(Li_1.0037_Fe_0.6962_Mn_0.0951_Al_1.293_)^Z^(Al_5.362_Si_0.638_)^T^[Si_5.0096_B_0.9904_O_18_]^B^[BO_3_]_3_^V^(OH)_4_. The mean empirical formula for the samples of dravitic composition is^X^(Na_0.6702_Ca_0.2951_K_0.0188_□_0.0159_)^Y^(Li_0.0046_Al_0.7272_Fe_0.0063_Mn_0.0021_Mg_2.6989_Cr_0.0148_Ti_0.0676_)^Z^(Al_5.88_Si_0.12_)^T^[Si_6_O_18_]^B^[BO_3_]_3_^V^(OH)_4_.

**Figure 7:**
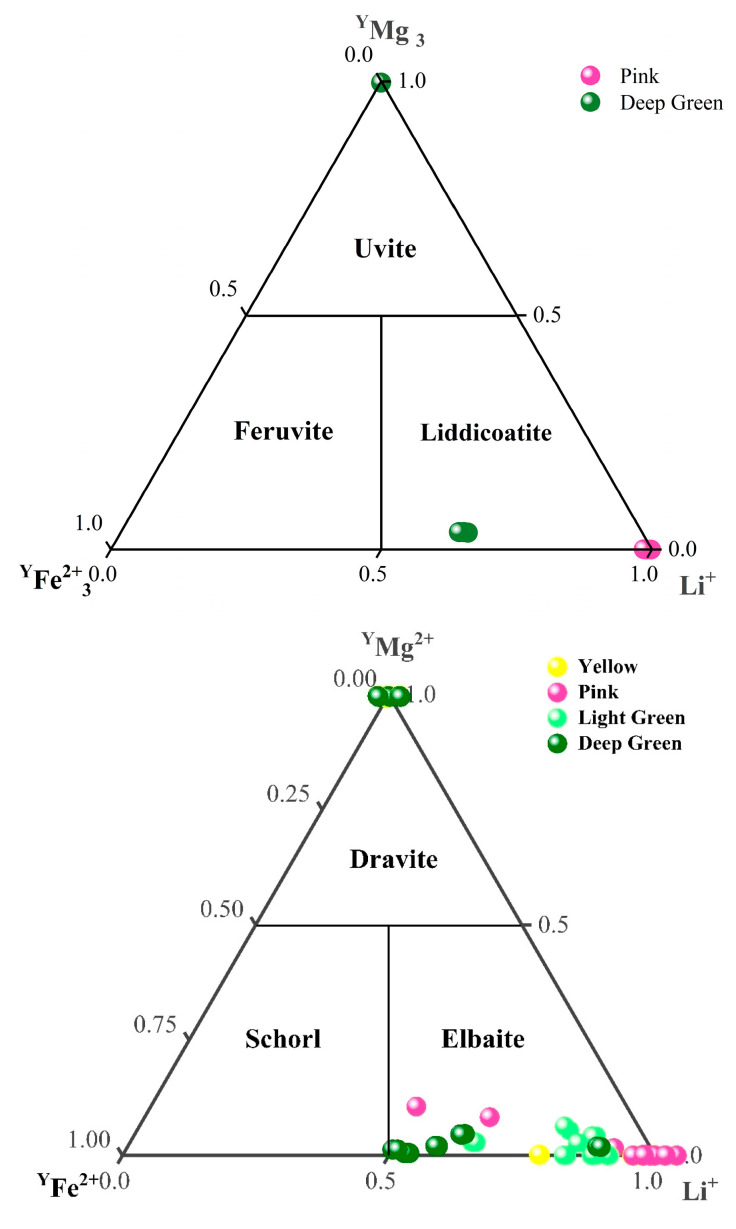
Y-site ternary plot. Each point represents the composition of a single sample determined by LA-ICP-MS analysis. The different colored samples are distinguished by colored labels. Data show that most of the tourmalines analyzed belong to the elbaite group.

The alkali tourmalines are further classified according to the contents of Li+Al, Mg, and Fe at the Y-site in a ternary plot [2]. Based on this classification, only 5 of the 32 samples tested are dravites, while the rest are of elbaitic composition (Figure 7). The samples show four colors: yellow, pink, light green, and dark green. The main gem-quality tourmaline locations in the world are China, the United States, Brazil, Zambia, Mozambique, Russia, the Congo, Nepal, Namibia, and more than a dozen of other regions, and the hue and color shades of tourmaline are mainly related to the three transition elements acting as color-causing agents: Mn, Ti, and Fe [14,24,25,26]. In the following section, the original compositional characteristics of the Yunnan tourmalines of different colors are analyzed by comparing the contents of specific trace elements with those of other tourmalines from different localities to provide information about their origin identification.

1. Yellow tourmalines

**Figure 8:**
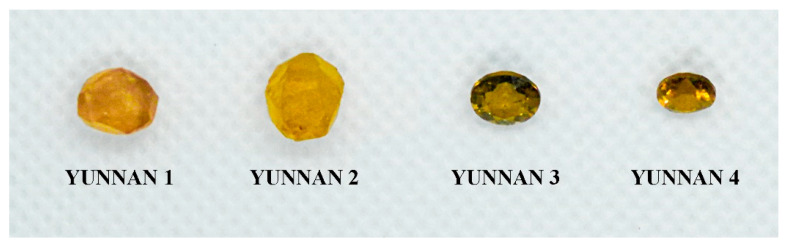
The four yellow tourmaline samples from Yunnan analyzed by LA-ICP-MS. The size of the gems is about 8–10 mm.

Four yellow tourmaline samples were analyzed by LA-ICP-MS (Figure 8), and the chemical composition for each sample was obtained using three spot analyses. The content of specific element oxides in wt% and average values are shown in Table 1. These yellow tourmalines are elbaite because they are (Al + Li) dominant. The contents of TiO_2_, MnO, FeO, and Li_2_O in the studied yellow tourmalines were compared with those of the same coloration from the production areas of Zambia, Russia, Madagascar, Nepal, the Congo, and California, and the average contents of TiO_2_, MnO, FeO, and Li_2_O for each production area were obtained from 35 compositional data points. The plots of TiO_2_ vs. MnO and TiO_2_ vs. FeO reporting all the data of Yunnan and other yellow tourmalines from different localities, are shown in Figure 8. The tourmaline samples from Yunnan are characterized by a higher Li_2_O content than those from other localities, with an average value of 1.90 wt%. The content of FeO is very low, comparable with that of tourmalines from Zambia, Madagascar, and California, whereas the contents of MnO andTiO_2_ are variable, oscillating between values of 1.42–6.41 wt% and 0.08 and 0.46 wt%, respectively, covering the compositional ranges of those from other localities. The TiO_2_, MnO, and FeO contents of Yunnan tourmaline span a wide distribution, and the interrelationships among the elements will be further discussed in subsequent research.

2. Green tourmalines

**Figure 9:**
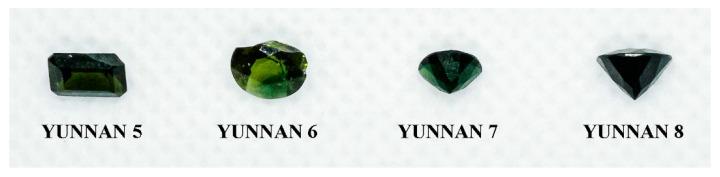
The four tourmaline samples from Yunnan analyzed by LA-ICP-MS. The size of the gems is about 8–11 mm.

Four tourmaline samples, green in color, were analyzed by LA-ICP-MS (Figure 9), and the chemical composition of each sample was obtained from three spot analyses. The content of specific element oxides in wt% and the relative average values are shown in Table 2.

### 2.4. Raman Spectrum Analysis

**Figure 10:**
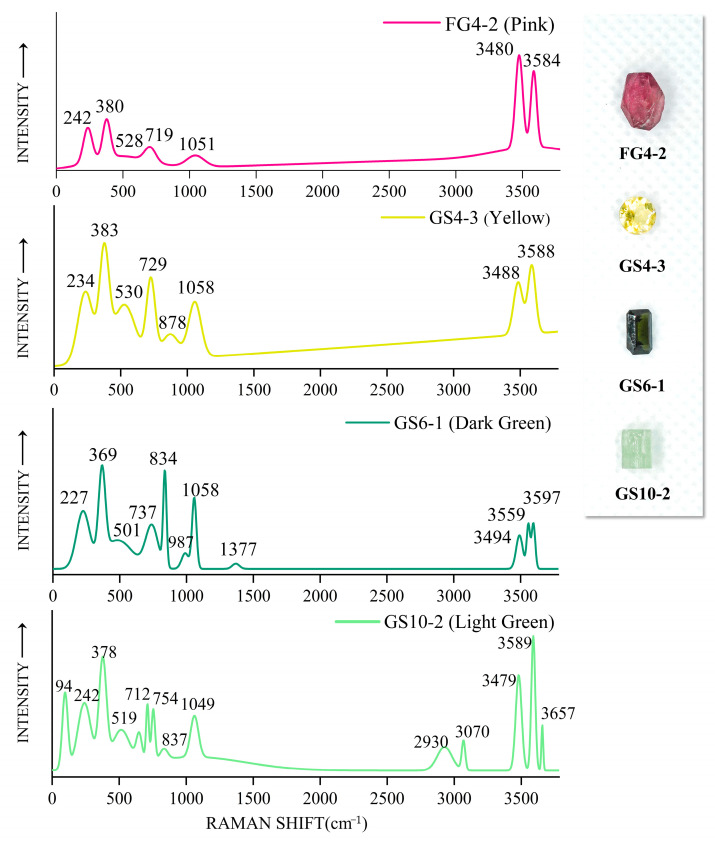
Raman spectra of tourmaline samples of different colors, with differences in indications depending on their elemental content.

The Raman spectra of the four colored samples (pink, yellow, green, and dark green) are shown in Figure 10. The peaks near 234 and 242 cm^−1^ are Mn/Fe-O with O-Al-O bending vibrations [11,12]; the peaks at 378 and 383 cm^−1^ are Al-O stretching vibrations; and the 519 cm^−1^ and 530 cm^−1^ Raman peaks belong to the symmetric vibrational mode of the [Si_6_O_18_]^12−^ hexagonal ring. The four colors differ significantly at the 835 cm^−1^ peak position, with the dark green GS6-1 vibrational peak being strong, the light green GS10-2 vibrational peak being weak, and the pink and yellow samples having no such peak. The vibrational peak near 835 cm^−1^ may be related to the appearance of Fe, which indicates that Fe dominates relative to Mn in this region [12]. The Si-O (non-bridging oxygen) stretching vibration peaks are located in the range of 1000–1200 cm^−1^ and the wave number is proportional to the SiO_2_ content, e.g., 1049 cm^−1^ for GS10-2 and 1058 cm^−1^ for GS4-3. The spectrum of the dark green sample shows that the peak value at 1377 cm^−1^ is the stretching vibration of C-O. The intensity of the spectral peak at 730 cm^−1^ is positively correlated with the B_2_O_3_ content [27]. The Raman peak of the B-O bond splits in two peaks when the symmetry of B atoms is reduced due to the different bond lengths of B and adjacent O [28]. This can explain the presence of the peaks at 712 and 754 cm^−1^ in the light green GS10-2 sample.

**Figure 11:**
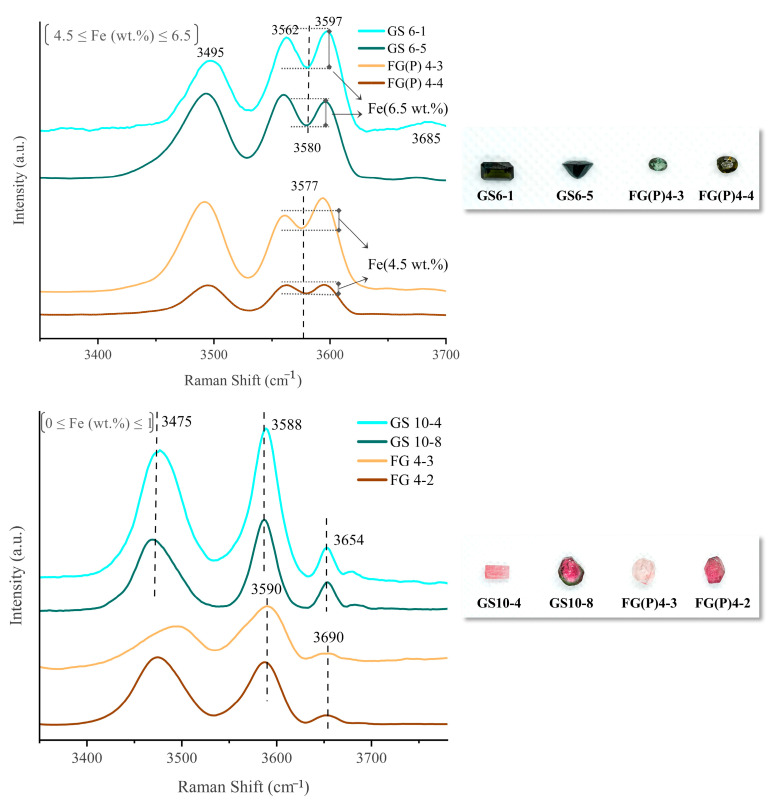
Influence of Fe content on OH vibrational peaks in the Yunnan tourmalines from the Fugong and Gongshan counties. The upper panel shows tourmalines with a high FeO content (range 6.5–4.5 wt.%), and the lower panel shows tourmalines with a low FeO content (below 1 wt.%), with a detection limit of 15 ppm for FeO measured by LA-ICP-MS. The extent of the Raman peaks varies depending on the Fe content.

Tourmaline typically has three [OH] Raman stretching vibrational peaks in the 3480–3670 cm^−1^ range, with the 3660 cm^−1^ Raman peak being attributed to the OH_1_ group in the hexagonal ring center and the two vibrational peaks near 3475 cm^−1^ and 3588 cm^−1^ being attributed to the OH_3_ in the hexagonal ring corner [29]. The Raman peak at the position of 2800–3000 cm^−1^ may be the Raman peak produced by the CH_4_-C_2_H_6_ gas mixture, as observed in sample GS10-2. Eight samples from the Gongshan and Fugong areas of Yunnan Province are selected for Raman spectroscopy; four represent the green color, and four represent the pink color (Figure 11). The Raman vibrational peak near 3580 cm^−1^ of the green tourmaline samples is split into two peaks at 3562 and 3597 cm^−1^, which is related to the high Fe content [11] (upper panel of Figure 11). The Raman peaks in the green tourmalines between 3650 and 3690 cm^−1^ are suppressed by the presence of Fe, showing a very weak intensity (upper panel of Figure 11). Combined with the Fe content revealed by the LA-ICP-MS analysis, it is seen that when the Fe content in the tourmaline samples reaches 6.5 wt.%, the splitting of the peak at 3580 cm^−1^ is greater (GS6-1 and GS6-5 samples) than that of the tourmaline samples with an FeO content of 4.5 wt% (FG 4-3 and FG 4-4 samples). Therefore, the degree of splitting of the peak at 3580 cm^−1^ is positively correlated with Fe in the samples; the higher the Fe content, the more significant the division.

### 2.5. UV-Vis Absorption Spectroscopy

**Figure 12:**
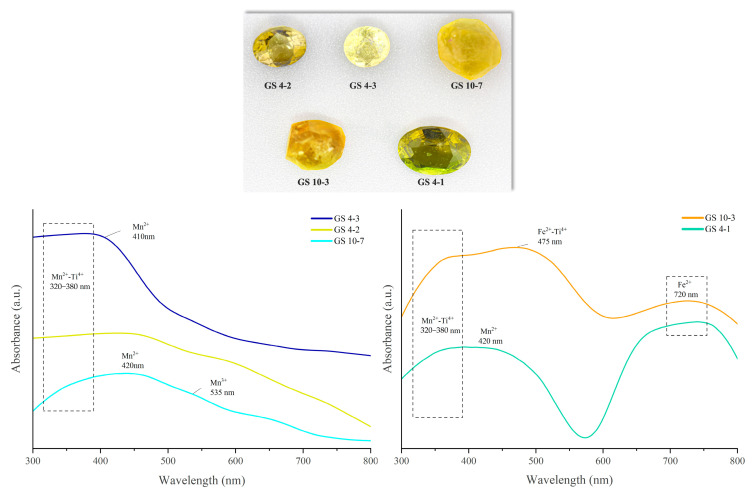
UV-vis absorption spectra of selected yellow tourmaline samples from Yunnan, where the left panel shows bright yellow tourmaline with Mn^2+^-Ti^4+^ as the main color-causing agent, and the right panel shows yellow-green tourmalines influenced by Fe^2+.^

The UV-Vis absorption spectra of the selected yellow tourmalines revealed that the color exhibited is mainly related to Mn^2+^ spin-forbidden transitions and Mn^2+^-Ti^4+^ IVCT transitions. Most of the selected samples (GS10-7, GS 10-3, and GS 4-1) show a broad absorption band in the visible region at about 320 nm due to Mn^2+^-Ti^4+^ IVCT transitions, causing absorption of the violet-to-blue wavelengths (Figure 12, left panel). Moreover, some samples (GS 4-3, GS 10-7, and GS 4-1) show a weak absorption band near 410 nm, which is caused by Mn^2+^ spin-forbidden transitions. The presence of this band enhances the absorption of the blue-violet wavelengths [12]. Additionally, Altieri et al., 2023, highlights that the yellow coloration of tourmaline crystals is caused by Mn^2+^, Mn^2+^, and Ti^4+^ interactions [25]. Sample GS 10-7 also shows a weak absorption band centered at 535 nm. This band is caused by Mn^3+^ d-d transitions and produces absorption in the yellow-green region of the spectrum, which shifts the yellow-toned tourmaline toward the brownish-yellow color [30]. It is interesting that sample GS 10-3 is also characterized by two broad absorption bands centered at 475 and 720 nm, which are caused by Fe^2+^-Ti^4+^ IVCT transitions and Fe^2+^ spin-allowed transitions, respectively [31] (Figure 12, right panel). The presence of Fe^2+^ caused the absorption of the orange-red wavelength and is also responsible for the greenish hue displayed by the sample. Consequently, the yellow-green hue displayed by some samples (GS10-3 and GS 4-1) is related to the combined effect of Mn^2+^, Ti^4+^, and Fe^2+^.

**Figure 13:**
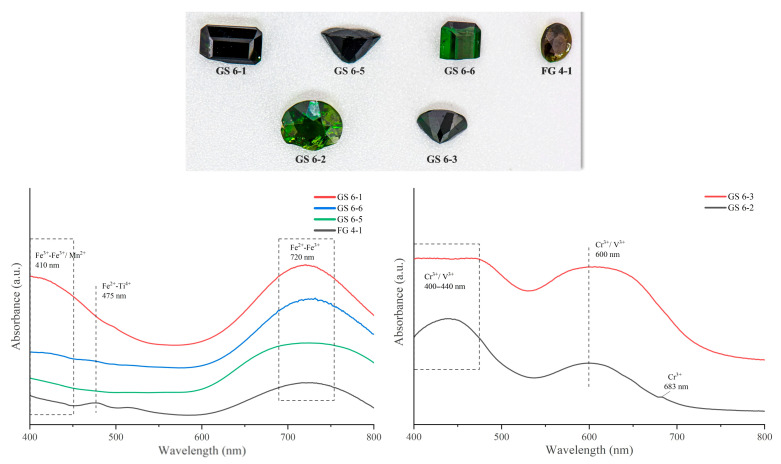
UV-vis absorption spectra of the green tourmalines from Yunnan. The left panel shows the tourmalines in which the green coloration is caused by Fe; the right panel shows the tourmalines in which the green coloration is due to Cr and V.

The visible absorption spectra of the selected green tourmalines show that Fe is the main color-causing agent responsible for the green coloration, as the FeO content in the selected samples reaches values up to 6 wt% according to chemical analysis. The broad absorption band centered at 410 nm can be caused by spin-forbidden Fe^2+^ and/or Fe^3+^ bands [32], causing absorption of the violet-to-blue wavelengths [33]. The samples contain Mn (Table 3), but its concentration is too low for it to be a color-causing agent. Sample FG 4-1 also shows a broad absorption band centered at 475 nm, which is caused by Fe^2+^-Ti^4+^ IVCT transitions (Figure 13, left panel). The wide absorption band centered at 720 nm is thought to be caused by electronic exchange interactions in an Fe^2+^-Fe^3+^ pair at adjacent Y-sites in the tourmaline structure, resulting in the absorption of the orange-red color of the spectrum [32]. In the GS 6-3 and GS 6-2 samples, the green coloration is instead caused by the presence of Cr^3+^ and V^3+^ (Figure 13, right panel). In fact, differently from the previously described greenish samples, samples GS 6-3 and GS 6-2 are characterized by absorption bands at about 440, 600 nm, and 680 nm, which are related to C^3+^ and V^3+^ spin-allowed transitions. Sample GS 6-2 is dominated by Cr^3+^, which caused strong absorption bands centered at 440 and 600 nm and a weak absorption band at 683 nm. Sample GS 6-3, which also contains Cr^3+^, is also characterized by higher levels of V^3+^, and forms an absorption peak centered at 440 nm [34].

**Figure 14:**
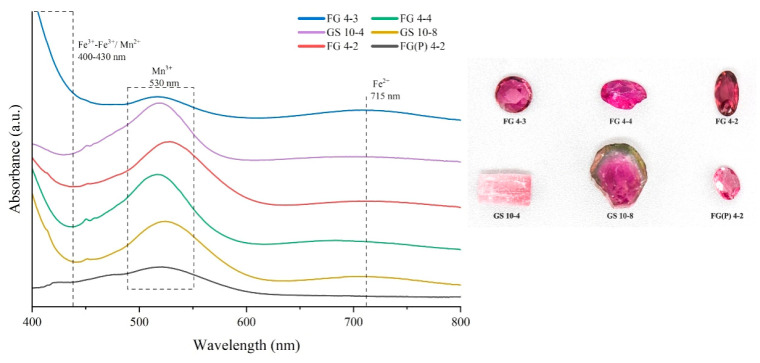
UV-vis absorption spectra of the Yunnan pink tourmalines indicating that the displayed color is mainly caused by Mn^3+^.

In the pink sample, the strong absorption band centered at 530 nm is caused by the d-d transition of Mn^3+^ (Figure 14). The weak absorption peak near 720 nm is associated with Fe^2+^ d-d transitions. The sample absorbs in the blue-violet, green, and red regions, respectively, with better transmission in the yellow and orange-red regions; consequently, these samples show a pinkish coloration [35].

### 2.6. Infrared Spectroscopy Analysis

**Figure 15:**
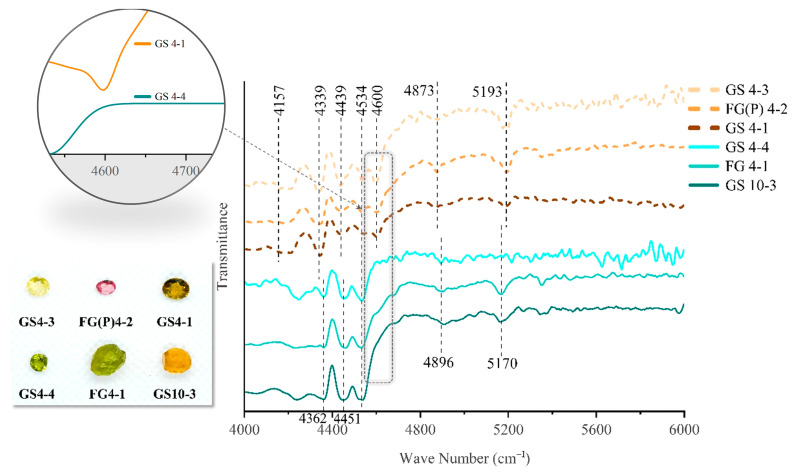
Infrared spectra of selected tourmaline analyzed of elbaitic and dravitic composition. The dashed yellow line refers to the elbaite composition; the solid blue line refers to the dravite composition. The 4000–4800 cm^−1^ range is the vibrational frequency of OH and metal cation combination, and 4600 cm^−1^ can be used as a quick differentiation between elbaite and dravite due to the difference in contents of Fe and Mg (as shown in the dotted box).

In the infrared spectrum of Yunnan tourmalines, 4000–4800 cm^−1^ is the frequency of the combination of Al, Mg, Fe, and a hydroxyl group. Different combinations of metal ions and hydroxyl groups will result in different performances in this band [36]. As shown in the general formula of tourmaline—XY_3_Z_6_(T_6_O_18_)(BO_3_)_3_V_3_W—the content of metal ions at the Y-site of the tourmaline structure varies significantly. The Y-site of elbaite usually occupied by about 65% of Al, and the remaining part by Li and Fe [2], respectively, and dravite at the Y-site has about 85% of Mg. Therefore, the IR spectra of elbaite and dravite from the same region of Yunnan are compared to verify the effect of different metal ions on the different ranges of the IR spectra. The spectra of Yunnan tourmaline in the near-infrared region of 4000–6000 cm^−1^ are shown in Figure 15.

The 4600 cm^−1^ position is thought to be due to Al-OH [37], and this absorption peak appears in tourmalines with elbaite composition, whereas the same absorption peak is completely missing in tourmalines with a dravitic composition due to the replacement of Al by Mg in dravite. So, the presence of the absorption band at 4600 cm^−1^ can be used to distinguish between elbaite and dravite species.

**Figure 16:**
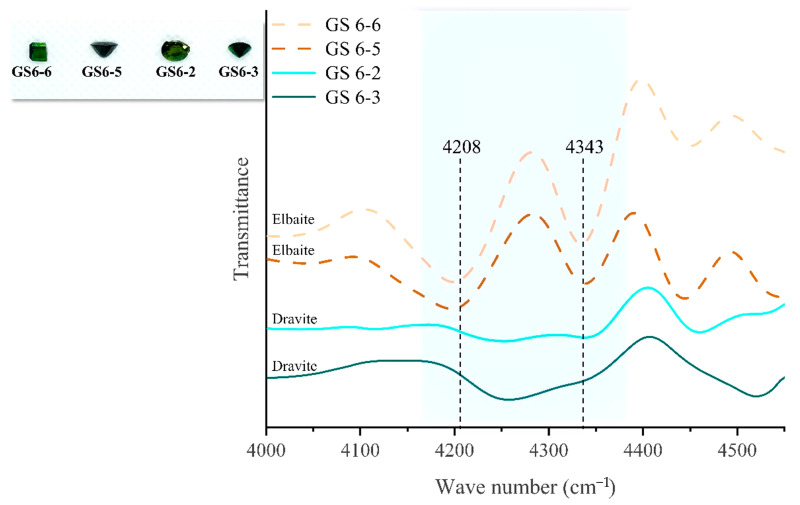
The dashed yellow line represents elbaite, and the solid blue line represents dravite. Due to the differences in their metal ions, when the vibration frequencies of OH and Fe ions are 4208 and 4343 cm^−1^ respectively, they can be used as a quick means to distinguish between ebaite and dravite (as shown in the blue area of the figure).

The absorption peaks in the range of 4300–4500 cm^−1^ are attributed to the interactions between Mg and Fe ions with OH. The combined frequency of [Mg-OH] is closer to 4500 cm^−1^ in the range of 4300–4500 cm^−1^, while [Fe-OH] contributes more to the vibrational peak near 4300 cm^−1^ [30]. The graph shows that 4534 cm^−1^ is present in both elbaite and dravite tourmaline species, and LA-ICP-MS data revealed that dravite has a greater intensity of the vibrational peak at 4534 cm^−1^ due to its Mg content reaching values of 10 wt.% of the total content, which is much higher than that of elbaite. To verify that the IR spectra near 4300 cm^−1^ are due to the co-relation of Fe with OH, two groups of elbaite and dravite of the same dark green hue from the Gongshan region were selected. The sample with elbaite composition has an absorption peak at 4343 cm^−1^, while dravite has no peak at this position (Figure 16). These results indicate that elbaite has a much higher iron content than dravite, and due to their identical sources, no significant IR shift was observed.

Elbaite species exhibit absorption peaks in the range of 4150–4200 cm^−1^ (Figure 15), which are supposed to be related to the combined Li-OH vibration.

The peak position corresponding to Si-O-Si is 4870 cm^−1^. There are two absorption peaks in the range of 4900–5400 cm^−1^ and 5193–5400 cm^−1^, which are generated by the bending and stretching vibrations of hydroxyl groups. This indicates that tourmaline is a water-based silicate.

### 2.7. Three-Dimensional Fluorescence Spectrum Analysis

**Figure 17:**
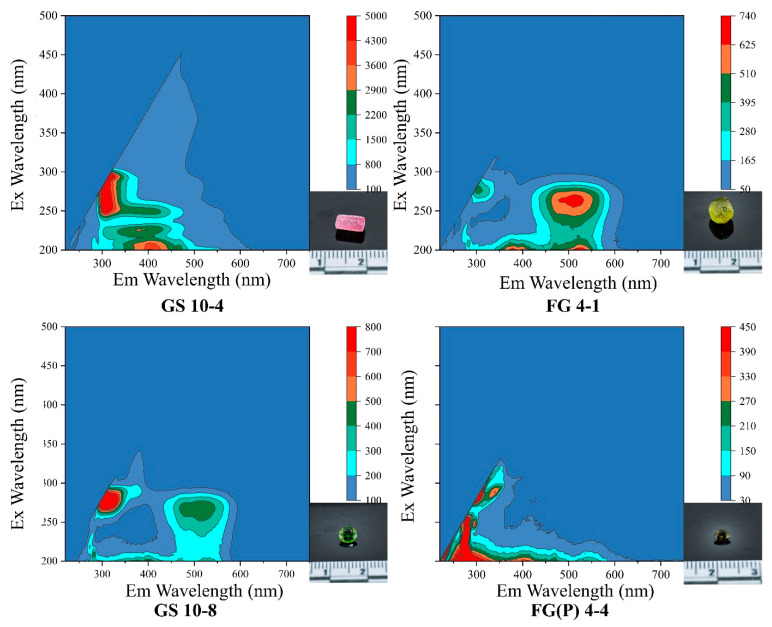
Performance of characteristic fluorescence peaks of multiple colored tourmalines under 3D fluorescence.

The samples exhibit fluorescence inert at long wavelengths and show blue-violet–weak yellow fluorescence at short wavelengths under a UV fluorescent light box. Selecting four Yunnan tourmaline samples and measuring the fluorescence peak position and optimal excitation wavelength under three-dimensional fluorescence, the fluorescence intensity was shown to be stronger from blue to red, and the energy was also correlated with the values on the right side of Figure 17.

Samples GS10-4, GS10-7, GS10-8, and FG4-4 all exhibit a characteristic fluorescence peak within the 3D fluorescence spectrum at λex280/λem320 nm with blue-violet fluorescence at short wavelengths; samples FG4-1 and FG10-8 also have fluorescence peaks at similar positions within the 3D fluorescence map, but these two tourmaline samples mentioned above also show another peak at λex265/λem510 nm at short wavelengths, with a light green fluorescence at short wavelengths.

The fluorescence of tourmaline samples is found to be correlated with the Fe and Ti contents measured by LA-ICP-MS [38]. The Fe and Ti contents of the selected samples are shown in Table 4. The weaker fluorescence of the FG(P)4-4 sample is clearly visible in the 3D fluorescence plots of the analyzed samples, inferring two general reasons: firstly, the content of Fe reached 4.10 wt.%, which is higher than that of the rest of the other samples analyzed, indicating that Fe has an inhibitory effect on the overall fluorescence effect; secondly, the sample size is too small, resulting in a weakened fluorescence effect. In the four samples, GS10-4 fluorescence is the strongest. The content of such sample is only 0.001 wt.%, which is lower than that observed in the other analyzed samples. The fluorescence of the sample is less suppressed, and the content of TiO_2_ reached 0.44 wt%, which is relatively high. This indicates that the sample has superior fluorescence intensity. The authors of [39] found a fluorescence peak at 496.5 nm at an excitation wavelength of 320 nm for TiO_2_ films, which is consistent with the content of Ti observed in the samples analyzed in this study, and they attributed it to the d-electron transitions of Ti^3+^ ions. In general, the fluorescence intensity of tourmaline is correlated with Fe and Ti contents [39].

### 2.8. Chemical Characteristics of This Provenance

1. Yellow tourmalines

**Figure 18:**
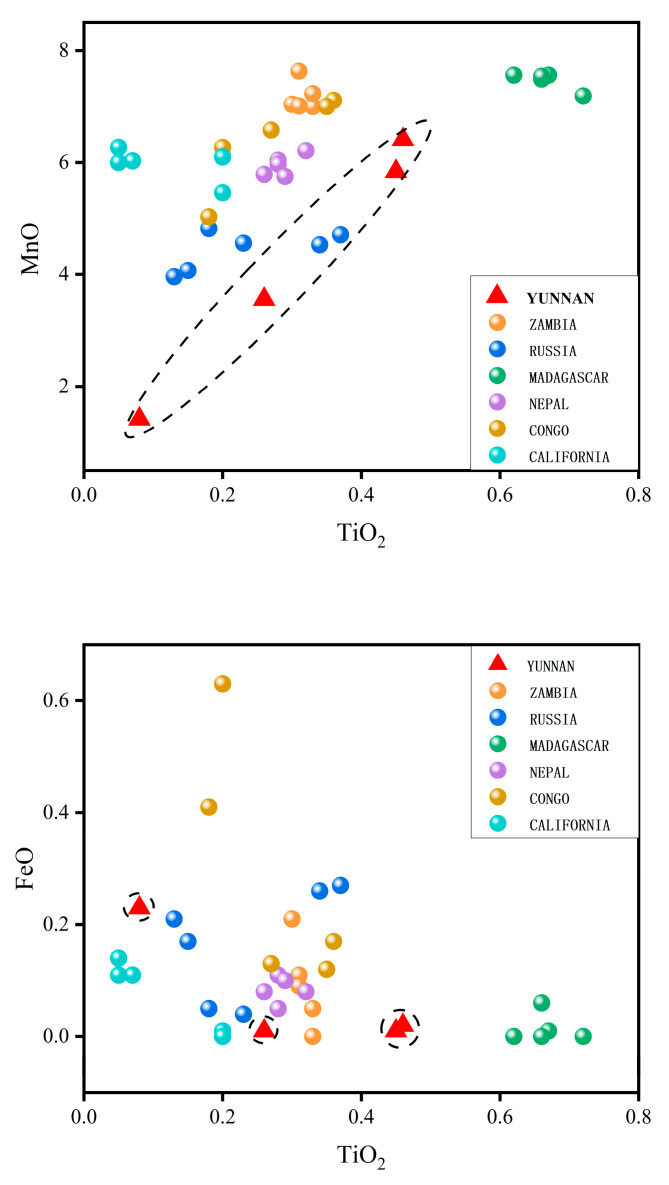
Plots of TiO_2_ vs. MnO and TiO_2_ vs. FeO. Dashed circle highlights the different distribution of the tourmalines from Yunnan Province.

As shown in Table 5, Madagascar and Zambian tourmalines exhibit high MnO and low FeO contents, with significantly higher MnO levels than Yunnan tourmalines, with the Madagascar sample having an average MnO content of 7.47 wt.%, the highest among the samples from the other localities and fluctuating in a stable range of 7.19–7.56 wt.%. The Madagascar samples have the highest TiO_2_ content among the samples from the other localities, being 0.67 wt.%, which is 2–3 times higher than the other localities. As shown in Figure 18, Zambian tourmaline has the next highest content of MnO, with an average value of 7.18 wt.%, while the Fe content is higher than that of the Madagascar tourmaline, with some samples overlapping with Congolese tourmaline in the scatter plot. The MnO content of Nepalese tourmaline has small fluctuations ranging from 5.75 to 6.21 wt.%, while the TiO_2_ content is more stable at a range of 0.26–0.32 wt.%, falling in the middle region of the various origins, similar to California tourmaline. Because the element content of Yunnan tourmaline fluctuates greatly, it can be distinguished from Yunnan tourmaline according to the density in the scatter diagram. Russian tourmaline has a low MnO content; some samples have a high FeO content, which fluctuates less; and some samples overlap with the Yunnan region in the scatter plot. The MnO content of the Congo tourmaline samples reaches 6.4 wt.%, and the FeO content has large fluctuations in the range of 0.12–0.63 wt.% with an average of 0.26 wt.%.

2. Green tourmalines

**Figure 19:**
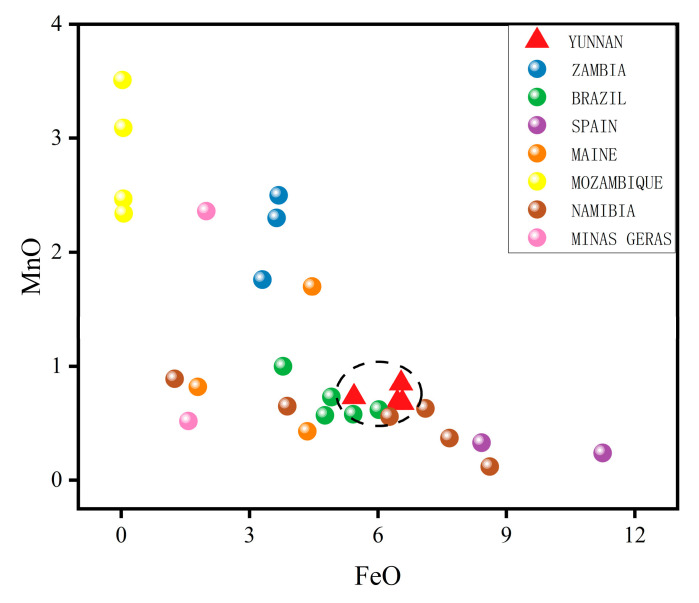
Plots of FeO vs. MnO. Dashed circle highlights the different distribution of the tourmalines from Yunnan Province.

The average values of TiO_2_, FeO, MnO, and Li_2_O of the green tourmalines from China were compared with those of 29 tourmaline samples with the same coloration from Zambia, Brazil, Maine, Minas Gerais, Mozambique, Namibia, and Brazil (Minas Gerais) (Table 6). By observing the plot of FeO vs. MnO (Figure 19), it can be observed that Yunnan tourmalines are concentrated in the middle-lower part of the plot, showing a high content of FeO (reaching an average value of 6.5 wt%) and a low content of MnO, which is below 1 wt%. The FeO and MnO contents of Yunnan tourmalines are quite similar to those of Brazilian tourmaline samples.

## 3. Materials and Methods

### 3.1. The Samples Studied

**Figure 20:**
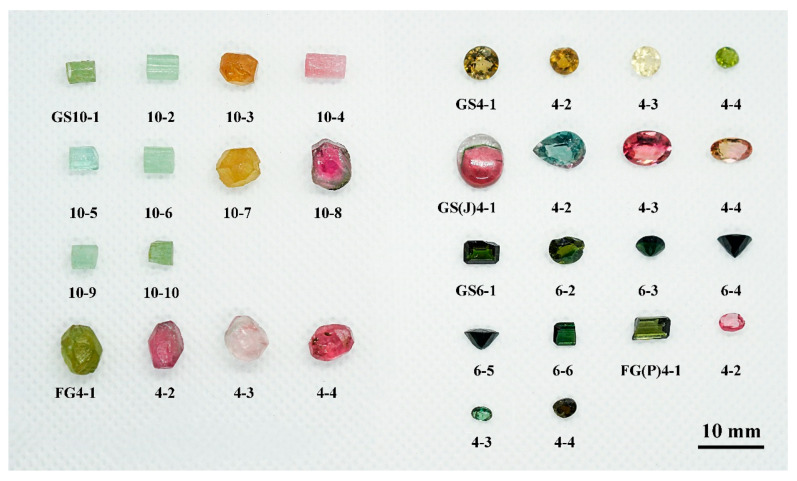
The studied tourmaline samples of various colors from Yunnan Province. The group on the left consists of unpolished tourmalines, and the group on the right consists of faceted tourmalines. The weights range from 0.2 to 2.5 ct. Photographed by Yu and Mingjun.

A total of 32 tourmaline samples from the Fugong and Gongshan counties and the surrounding areas of Yunnan Province (China) were studied. The studied samples include 18 faceted stones and 14 unpolished rough stones (Figure 20). The colors of the samples range from yellow to pink, dark green, and light green, with a transparent to translucent and glassy luster.

The tourmaline samples analyzed in this work are from the Yunnan Tourmaline Museum, founded by Mr. Lan Huiming, which is the largest tourmaline museum in China. The manuscript also incorporates tourmaline samples from other localities.

### 3.2. Experimental Set-Up

Fourier-transform infrared spectra were created using a Bruker Vertex80 spectrometer (Bruker, Billerica, MA, USA) at the School of Jewelry, China University of Geosciences (Wuhan, China). Spectra were obtained in transmission mode, with an aperture of 8 mm, a scanning frequency of 10 kHz, a resolution of 4 cm^−1^, and a scanning range of 6000 to 2000 cm^−1^. Laser Raman spectroscopy was performed at the State Key Laboratory of Biogeology and Environmental Geology, China University of Geosciences (Wuhan, China), using an Alpha 300-R Raman spectrometer (Witec, Ulm, Germany). A 532 nm laser was used as an excitation light source, with a scanning time of 20 s, one superposition, a scanning range of 0–4000 cm^−1^, and an aperture of 50 μm for the diaphragm. The LA-ICP-MS test was performed at Wuhan SampleSolution Analytical Technology Co., Ltd. (Wuhan, China), using Agilent 7900 (Agilent Technologies, Santa Clara, CA, USA); the carrier gas was helium, with a flow rate of 650 mL/min, and the RF power was 1500, and the tool was equipped with a laser beam spot of 44 μm in diameter, a laser frequency of 5 Hz, and an energy density of 5.5 J·cm^−2^. The ablation laser was a 193 ns excimer laser, and the calibration equipment included four USGS glass specimens: NIST 610, BCR-2G, BHVO-2G, and BIR-1G. Three spots were tested for each sample, with a 40 s dwell time per point; UV-Vis spectroscopy was performed at the School of Jewelry, China University of Geosciences (Wuhan, China), using a Perkin Elmer Lambda650S (PerkinElmer, Waltham, MA, USA). Spectra were collected in the range of 350–780 nm, with a resolution of 1 nm, in transmission mode to test the contents of chromogenic elements in the sample. Three-dimensional fluorescence spectroscopy was performed with a Japanese Jasco FP8500 fluorescence spectrometer (JASCO, Hachioji, Japan) at the School of Jewelry, China University of Geosciences (Wuhan, China), with the excitation spectrum range set to 200–500 nm and the bandwidth of the excitation spectrum set to 5 nm, and the data interval was 2 nm. The range of the emission spectra was set in a range of 220–750 nm, the bandwidth of the emission spectra was set to 2.5 nm, and the data interval was 1 nm. A Leica M205A/DFC550 microscope (Leica Microsystems, Wetzlar, Germany) was used to photograph the sample contents with an irradiation source of 5500 K.

## 4. Conclusions

This study provides the first detailed characterization of the gemological properties, chemical composition, and spectroscopic properties of Yunnan tourmalines from the Nujiang River coast, China. Based on the LA-ICP-MS results, a compositional analysis of Yunnan tourmalines with dozens of reported yellow and green tourmalines from other localities was performed, and the results show that Yunnan tourmalines are characterized by a wide range of variation in major color-causing elements such as Mn/Fe/Ti/Cr. Raman and infrared spectra together confirm the presence of Fe and Mg ions. The influence of Fe on the absorption vibrations observed at 835 cm^−1^ and 3580 cm^−1^ was examined. The incorporation of Fe produces a characteristic Raman peak at 835 cm^−1^ and induces varying degrees of splitting of the Raman peak near 3580 cm^−1^, which correlates with the Fe content. Yunnan Province yields tourmalines with both elbaite and dravite compositions, where infrared spectra exhibiting peaks at 4600 cm^−1^, 4343 cm^−1^, and 4200 cm^−1^ allow rapid differentiation between these two species. UV-vis absorption spectra demonstrate that bright yellow coloration stems from Mn^2+^-Ti^4+^ charge-transfer transitions, while the presence of Fe induces a shift toward yellow-green hues. Green coloration primarily results from Fe, though Cr and V contribute in specific cases. Pink hues are attributed to Mn^3+^ in select specimens. Three-dimensional fluorescence showed that, in addition to the conventional λex280/λem320 nm main fluorescence peak, some samples showed additional λex265/λem510 nm fluorescence peaks, and the causes and intensities of fluorescence were related to the Ti and Fe contents. Collectively, these findings position Yunnan as a prominent source of high-quality tourmalines with respect to their rich diversity of colors and overall quality.

## Figures and Tables

**Table 1:** Summary of selected element oxides (wt.%) characterizing the yellow tourmalines from Yunnan. For each sample, the composition of 3 spot analyses was considered.

	Li_2_O	TiO_2_	FeO	MnO
Range	1.72–2.41	0.08–0.46	0.01–0.23	1.42–6.41
Average	1.90	0.31	0.07	4.31
Median	1.74	0.355	0.02	4.52
Detection limits (wt.%)	0.0002–0.0004	0–0.0006	0.004–0.005	0.0002–0.0003

**Table 2:** Summary of selected element oxides (wt.%) which characterize the green tourmalines from Yunnan. For each sample, the composition of 3 spot analyses was considered.

	Li_2_O	TiO_2_	FeO	MnO
Range	1.40–1.62	0.03–0.13	6.44–6.57	0.68–0.85
Average	1.53	0.06	6.25	0.74
Median	1.54	0.03	6.495	0.71
Detection limits (wt.%)	0.0002–0.0005	0–0.0005	0.004–0.006	0.0002–0.0004

**Table 3:** Contents of different chromophore elements in the green tourmalines.

Number/Element (wt.%)	LiO_2_	Cr_2_O_3_	V_2_O_5_	FeO	MnO	TiO_2_
GS 6-1	1.62	0.0002	bdl	5.44	0.73	0.13
GS 6-2	0.004	0.15	0.06	0.04	0.004	0.62
GS 6-3	0.021	0.37	1.02	0.02	0.007	0.54
GS 6-4	1.54	bdl	bdl	6.35	0.67	0.03
GS 6-5	1.54	0.0007	bdl	6.57	0.68	0.03
FG 4-1	2.57	bdl	bdl	1.38	0.45	0.05
Detection limits (wt.%)	0–0.0002	0.0006–0.0012	0–0.00002	0.0033–0.0052	0.0001–0.0003	0–0.0004

**Table 4:** Comparison of Fe/Ti contents of different colored tourmalines.

Sample Number	TiO_2_ (wt.%)	FeO (wt.%)
GS10-4	0.005	0.001
FG4-1	0.57	0.01
GS 10-8	0.002	0.10
FG(P)4-4	0.09	4.10
Detection limits (wt.%)	0–0.0005	0.004–0.005

**Table 5:** Mean values of selected element oxides (in wt%) in the Yunnan yellow tourmalines and in tourmaline samples from different localities.

	Yunnan	Zambia *	Russia *	Madagascar *	Nepal *	Congo *	California *
TiO_2_	0.31	0.32	0.23	0.67	0.29	0.27	0.11
FeO	0.07	0.09	0.17	0.01	0.08	0.29	0.07
MnO	4.31	7.18	4.44	7.47	5.95	6.40	5.97
Li_2_O	1.90	1.30	1.79	1.52	1.42	1.69	1.50

* Tourmaline samples from these localities were reported in [40]. The data in this table are average values of selected element oxides for each considered locality. Not all data are reported.

**Table 6:** Mean values of selected element oxides (in wt%) in the Yunnan green tourmalines and in tourmaline samples from different localities.

Element (wt.%)/Origin	Yunnan	Zambia ^a^	Brazil ^b^	Maine ^c^	Mozambique ^d^	Namibia ^e^	Minas Gerais ^f^
TiO_2_	0.06	0.05	0.21	0.09	0.05	0.06	0.02
FeO	6.25	3.54	4.98	3.53	0.05	5.80	1.78
MnO	0.74	2.19	0.70	0.98	2.85	0.54	1.44
Li_2_O	1.53	1.77	1.53	1.54	1.22	1.29	2.60

^a^ Zambian samples [41]. ^b^ Brazilian samples [42]. ^c^ MAINE samples [40]. ^d^ Mozambican samples [7]. ^e^ Namibian samples [23]. ^f^ Minas Gerais State samples [23]. The data in this table are the average values of selected element oxides for each considered locality. Not all data are reported.

## Data Availability

The data presented in the study are available in the article.

## References

[1] Henry D.J., Dutrow B.L. (2018). Tourmaline studies through time: Contributions to scientific advancements. J. Geosci..

[2] Henry D.J., Novák M., Hawthorne F.C., Ertl A., Dutrow B., Uher P., Pezzotta F. (2011). Nomenclature of the tourmaline supergroup minerals. Am. Min..

[3] El-Hinnawi E., Hofmann R. (1966). Optische und chemische Untersuchungen an neun Turmalinen (Elbaiten). Neues Jahrb. Mineral..

[4] Katsurada Y., Sun Z., Breeding C.M., Dutrow B.L. (2019). Geographic origin determination of paraíba tourmaline. Gems Gemol..

[5] Okrusch M., Ertl A., Schüssler U., Tillmanns E., Brätz H., Bank H. (2016). Major-and trace-element composition of paraíba-type tourmaline from brazil, mozambique and nigeria. J. Gemmol..

[6] Abduriyim A., Kitawaki H., Furuya M., Schwarz D. (2006). “Paraiba”-type copper-bearing tourmaline from Brazil, Nigeria, and Mozambique: Chemical fingerprinting by LA-ICP-MS. Gems Gemol..

[7] Ertl A., Giester G., Schüssler U., Brätz H., Okrusch M., Tillmanns E., Bank H. (2013). Cu-and Mn-bearing tourmalines from Brazil and Mozambique: Crystal structures, chemistry and Correlations. Mineral. Petrol..

[8] Roda-Robles E., Pesquera A., Gil P., Torres-Ruiz J., Fontan F. (2004). Tourmaline from the rare-element Pinilla pegmatite, (Central Iberian Zone, Zamora, Spain): Chemical variation and implications for pegmatitic evolution. Mineral. Petrol..

[9] Simmons W.B., Laurs B.M., Falster A.U., Koivula J.I., Webber K.L. (2005). Mt. Mica: A Renaissance in Maine’s Gem Tourmaline Production. Gems Gemol..

[10] Zhong R. (2014). Gem Mineralogical Characteristics of Blue-Green Tourmaline in Nurstan, Afghanistan. Master’s Thesis.

[11] Hoang L.H., Hien N.T.M., Chen X.B., Minh N.V., Yang I.S. (2011). Raman spectroscopic study of various types of tourmalines. J. Raman Spectrosc..

[12] Laurs B.M., Simmons W.B., Rossman G.R., Fritz E.A., Koivula J.I., Anckar B., Falster A.U. (2007). Yellow Mn-rich tourmaline from the Canary mining area, Zambia. Gems Gemol..

[13] Prasad P.S.R. (2005). Study of structural disorder in natural tourmalines by infrared spectroscopy. Gondwana Res..

[14] Suwanmanee W., Wanthanachaisaeng B., Utapong T., Sutthirat C. (2021). Colour Enhancement of Pink Tourmaline from Nigeria by Electron-Beam and Gamma Irradiation. J. Gemmol..

[15] Watenphul A., Burgdorf M., Schluter J., Horn I., Malcherek T., Mihailova B. (2016). Exploring the potential of Raman spectroscopy for crystallochemical analyses of complex hydrous silicates: II. Tourmalines. Am. Mineral..

[16] Peng Z.S. (1992). Geological background and main characteristics of tin ore in the Nu River area. Geol. Explor..

[17] Bu X.F. (2014). Geochemistry, Chronology and Spatiotemporal Evolution of Granitoids in Nujiang Area, Northwest Yunnan Province. Master’s Thesis.

[18] Liu J. (2017). Gemological Characteristics of Tourmaline and Its Internal Inclusions. Master’s Thesis.

[19] Zeng X.Y. (2016). CH_4_/CH_4_-C_2_H_6_ Hydrate Formation and Decomposition in Situ Raman Studies. Ph.D. Thesis.

[20] Zerda T.W., John A., Chmura K. (1981). Raman studies of coals. Fuel.

[21] Zhang N., Tian Z.J., Mao G.J., Wu S.H., Liu J.X., De Q. (2009). Raman spectral characteristics of asphalt inclusions. Geochemistry.

[22] Liu G.B., Lu H.Z. (1985). Study on inclusions in gem-grade colored tourmaline in Xinjiang. Geochemistry.

[23] da Fonseca-Zang W.A., Zang J.W., Hofmeister W. (2008). The Ti-influence on the tourmaline color. J. Braz. Chem. Soc..

[24] Altieri A., Pezzotta F., Skogby H., Hålenius U., Bosi F. (2022). Blue-growth zones caused by Fe^2+^ in tourmaline crystals from the San Piero in Campogem-bearing pegmatites, Elba Island, Italy. Mineral. Mag..

[25] Altieri A., Pezzotta F., Skogby H., Hålenius U., Bosi F. (2023). Dark-coloured Mn-rich overgrowths in an elbaitic tourmaline crystal from the Rosina pegmatite, San Piero in Campo, Elba Island, Italy: Witness of late-stage opening of the geochemical system. Mineral. Mag..

[26] Hawthorne F.C., Henry D.J. (1999). Classification of the minerals of the tourmaline group. Eur. J. Mineral..

[27] Brethous J.C., Levasseur A., Villeneuve G., Echegut P., Hagenmuller P., Couzi M. (1981). Etudes par spectroscopie Raman et par RMN des verres du système B_2_O_3_ SiO_2_ Li_2_O. J. Solid State Chem..

[28] Fu X.M. (1998). Polarized Raman spectroscopy of tourmaline. Miner. Geol..

[29] Wesełucha-Birczyńska A., Natkaniec-Nowak L. (2011). A Raman microspectroscopic study of organic inclusions in “watermelon” tourmaline from the Paprok mine (Nuristan, Afghanistan). Vib. Spectrosc..

[30] Reddy B.J., Frost R.L., Martens W.N., Wain D.L., Kloprogge J.T. (2007). Spectroscopic characterization of Mn-rich tourmalines. Vib. Spectrosc..

[31] Wang J.J., Tao X.F., Wang W.J. (2005). Study on the color genesis of green tourmaline in Xinjiang. J. Rock Mineral..

[32] Mattson S.M., Rossman G.R. (1987). Fe^2+^-Fe^3+^ interactions in tourmaline. Phys. Chem. Miner..

[33] Pasetti L., Borromeo L., Bersani D., Andò S., Schnellrath J., Hennebois U., Karampelas S. (2023). Identification of Some Gem Quality Blue to Green Li-Tourmalines. Minerals.

[34] Rondeau B., Fritsch E., Peucat J.-J., Nordrum F.S., Groat L. (2008). Characterization of emeralds from a historical deposit: Byrud (Eidsvoll), Norway. Gems Gemol..

[35] Fan J.L., Feng X.Q., Guo S.G., Liu X.L. (2009). Optical absorption spectra of tourmaline crystals. J. Chin. Ceram. Soc..

[36] Surour A.A., Omar S.M.A. (2022). Chemical and spectroscopic characterization of tourmaline from the ancient Roman mines in the Eastern Desert of Egypt. Environ. Earth Sci..

[37] Li X.J., Zu E.D. (2016). Near-infrared spectroscopy of cyclic silicate gem minerals. Bull. Silic..

[38] Liao Q.J., Huang W.Z., Zhang Q., Pei J.C. (2019). Gemological and spectroscopic characterization of brown-yellow tourmaline in Mozambique. Spectrosc. Spectr. Anal..

[39] Liu B.S., He X., Zhao J.J., Zhao Q.N. (2005). Effects of heat treatment on structure and spectral properties of TiO_2_ sputtered thin films. Rare Met. Mater. Eng..

[40] Simmons W.B., Falster A.U., Laurs B.M. (2011). A survey of Mn-rich yellow tourmaline from worldwide localities and implications for the petrogenesis of granitic pegmatites. Can. Mineral..

[41] Zhong P.P., Shen X.T. (2017). A preliminary study on the color genesis of dark green tourmaline in Zambia. J. Gems Gemol..

[42] Da Silva S.F., Moura M.A., Queiroz H.d.A., Ardisson J.D. (2018). Chemical and spectroscopic characterization of tourmalines from the Mata Azul pegmatitic field, Central Brazil. J. Geosci..

